# Best evidence summary of digital health interventions for self-management in patients with coronary artery disease

**DOI:** 10.3389/fpubh.2026.1833261

**Published:** 2026-06-19

**Authors:** Xinyan Chen, Haiyan Zheng, Yuxin Han, Xiyao Yang, Xinyu Wu, Zhilian Zhang, Wei Deng, Sha Yue, Lixiong Bi, Guilan Zhang, Yongyong Ding

**Affiliations:** 1 Yunnan University of Chinese Medicine, Kunming, Yunnan, China; 2 Kunming Hospital of Traditional Chinese Medicine, Kunming, Yunnan, China

**Keywords:** coronary artery disease, digital health intervention, evidence-based nursing, health behavior change, self-management

## Abstract

**Objective:**

To systematically identify and synthesize the best available evidence on digital health interventions supporting self-management in patients with coronary artery disease (CAD). The aim of this study was to provide evidence-based guidance for healthcare professionals and to inform the development of digital intervention–based self-management programs that can support patients in optimizing their self-management behaviors and practices.

**Methods:**

A comprehensive evidence summary was conducted in accordance with the methodology recommended by the Joanna Briggs Institute (JBI). Systematic searches were performed in PubMed, Embase, Web of Science, CINAHL, the Cochrane Library, and major Chinese databases from their inception to November 2025. The methodological quality of the included studies was appraised using JBI critical appraisal tools. The certainty of evidence and strength of recommendations were evaluated using the GRADE approach.

**Result:**

s**:** A total of 5,312 records were initially identified, of which 38 studies met the inclusion criteria. These included six clinical guidelines, 13 systematic reviews, 15 randomized controlled trials, and four quasi-experimental studies. From these sources, 36 pieces of evidence were synthesized and categorized into five domains: intervention modalities, self-management support strategies, medication adherence, lifestyle management, and clinical outcomes.

**Conclusions:**

This evidence summary provides a comprehensive synthesis of digital health interventions designed to enhance self-management among patients with CAD. The findings offer a scientific basis for healthcare professionals to integrate digital health strategies into clinical practice and highlight their potential role in strengthening self-management and improving health outcomes in this population.

**Systematic Review Registration:**

https://www.crd.york.ac.uk/PROSPERO/view/CRD420251228969, Identifier: CRD420251228969.

## 1 Introduction

Coronary artery disease (CAD) remains one of the leading causes of mortality and disability worldwide (1). It is primarily caused by atherosclerotic narrowing or occlusion of the coronary arteries, which results in myocardial ischemia or hypoxia and may lead to severe complications such as heart failure and sudden cardiac death (2, 3). With the ongoing global trends of population aging and the increasing prevalence of major cardiovascular risk factors—including hypertension, diabetes mellitus, and obesity—the burden of CAD continues to rise (4, 5). Epidemiological evidence indicates that CAD accounts for approximately 76 deaths per 100,000 population, and projections suggest that by 2050 it will become the most prevalent cardiovascular disease globally, affecting an estimated 471 million individuals and contributing to a 93.4% increase in mortality (6).

In clinical practice, the management of CAD typically involves pharmacological therapy, percutaneous coronary intervention (PCI), and coronary artery bypass grafting (CABG). However, CAD is a chronic condition characterized by a prolonged disease trajectory, and effective management often extends beyond hospital care. After discharge, patients frequently encounter challenges in maintaining long-term self-management, including poor medication adherence and difficulties sustaining lifestyle modifications. These issues can significantly compromise clinical outcomes and increase the risk of recurrent cardiovascular events (7, 8). Therefore, effective interventions are required to enhance patients' self-management capabilities, promote sustainable health behavior changes, and ultimately improve quality of life and long-term prognosis. Such strategies are particularly important for addressing the limitations of traditional healthcare models and empowering patients to take a more active role in disease management.

Digital health interventions (DHIs), which are based on information and communication technologies, have emerged as an important evidence-based approach for supporting health management. According to the World Health Organization, DHIs refer to the application of digital technologies to achieve specific health objectives, including mobile health (mHealth), remote monitoring systems, wearable devices, mobile applications, and web-based platforms (9, 10). As an innovative model for chronic disease management, DHIs can provide personalized guidance, real-time monitoring, and continuous feedback, thereby improving the acceptability and sustainability of self-management strategies among patients (11–14). Previous studies have demonstrated that digital health interventions can enhance medication adherence, improve health literacy, and strengthen self-management capacity in individuals with CAD (15–17).

In both developed and developing countries, the clinical application of digital health technologies is expanding rapidly. Several countries and regions, including Europe, Canada, and Australia, have introduced policies encouraging the integration of digital health strategies into the long-term management of cardiovascular diseases (18–20). Although a growing body of research has examined the use of digital interventions in cardiac rehabilitation and remote monitoring, comprehensive syntheses of the best available evidence specifically focusing on digital health interventions that support self-management in patients with CAD remain limited. Existing systematic reviews tend to focus primarily on cardiac rehabilitation programs or remote monitoring technologies, with relatively little emphasis on the broader role of DHIs in enhancing self-management capacity among individuals with CAD (21). Therefore, this study aims to systematically summarize the best available evidence regarding digital health interventions designed to support self-management in patients with coronary artery disease, thereby providing evidence-based guidance for clinical practice and nursing management.

## 2 Methods

### 2.1 Literature search strategy

This study was conducted as a best evidence summary following the methodology proposed by the Joanna Briggs Institute (JBI) for evidence synthesis. A comprehensive literature search was conducted to identify the best available evidence on digital health interventions for self-management in patients with coronary artery disease (CAD). The literature search followed the “6S” evidence pyramid model, prioritizing higher levels of evidence. Clinical decision support systems and clinical practice guidelines were searched first, followed by systematic reviews and meta-analyses. When higher-level evidence was insufficient, original research studies were subsequently retrieved.

**Figure 1:**
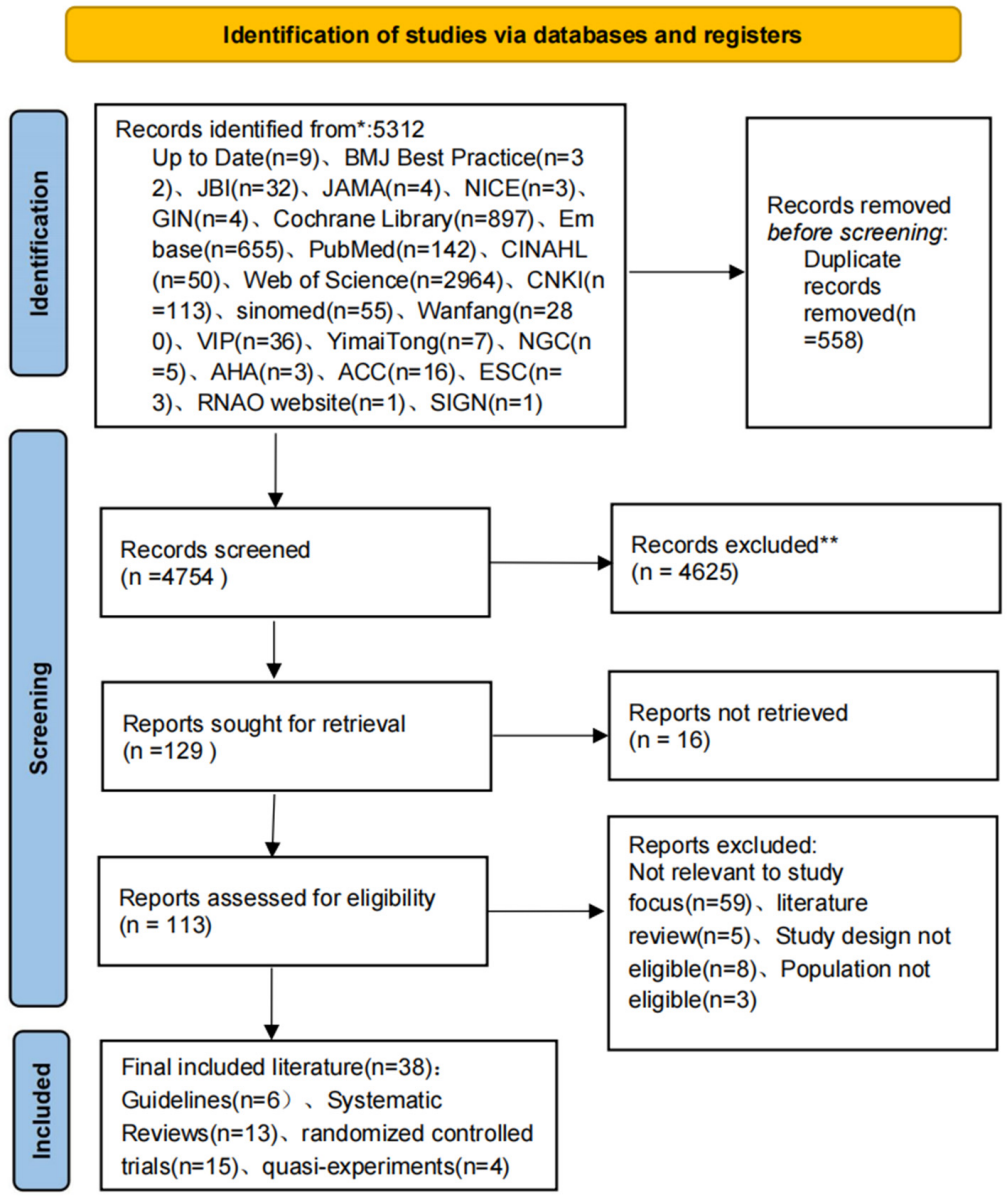
PRISMA flow diagram showing the identification, screening, eligibility assessment, and inclusion of studies.

Search terms combined concepts of (1) coronary artery disease, including “coronary artery disease,” “coronary heart disease,” “coronary atherosclerotic heart disease,” “ischemic heart disease,” “acute coronary syndrome,” “angina pectoris,” and “myocardial infarction”; (2) self-management, including “self-management,” “self-care,” “self-monitoring,” “self-care management,” and “health management”; and (3) digital health interventions, including “digital health,” “digital medicine,” “mobile health,” “mHealth,” “digital therapeutics,” “wearable devices,” “telemedicine,” and “remote monitoring.” The study selection process was conducted in accordance with the Preferred Reporting Items for Systematic Reviews and Meta-Analyses (PRISMA) guidelines, and the detailed process is presented in Figure 1.

Evidence sources included clinical decision support tools, guidelines, systematic reviews, and primary research databases, specifically: UpToDate, BMJ Best Practice, Joanna Briggs Institute, JAMA, National Institute for Health and Care Excellence, Guidelines International Network, Cochrane Library, Embase, PubMed, CINAHL, Web of Science, as well as Chinese-language databases including CNKI, SinoMed, Wanfang Data, VIP Database, and YimaiTong. In addition, professional organizations and guideline repositories were searched, including American Heart Association, American College of Cardiology, European Society of Cardiology, Registered Nurses' Association of Ontario, and Scottish Intercollegiate Guidelines Network.

All searches were independently performed by two reviewers to ensure completeness and reduce selection bias. The search period covered publications from database inception to November 2025. This systematic evidence summary was prospectively registered on the international systematic review registry PROSPERO (registration number: CRD420251228969).

### 2.2 Study selection

Inclusion Criteria: studies were eligible for inclusion if they met the following criteria: (1) participants were diagnosed with coronary artery disease (CAD); (2) the study addressed self-management, self-care, self-monitoring, or health management behaviors related to CAD; (3) interventions were digital health–based; (4) study types included clinical decision support tools, systematic reviews, guidelines, expert consensus statements, evidence summaries, and original research; and (5) publications were in English or Chinese.

Exclusion Criteria: studies were excluded if they met any of the following: (1) duplicate publications; (2) full texts were not accessible; (3) outdated guidelines or evidence that had been superseded by updated versions; or (4) publications that were guideline interpretations, conference abstracts, book reviews, commentaries, or articles without primary data.

All references were independently screened by two reviewers in a staged process according to the pre-defined inclusion and exclusion criteria. Discrepancies between reviewers were resolved through discussion with a third reviewer who had extensive experience in evidence-based practice and methodology. Duplicate records were removed using EndNote software.

### 2.3 Quality assessment of the included studies

#### 2.3.1 Quality assessment procedure

All included studies were independently appraised by two researchers trained in evidence-based nursing. Each researcher conducted an initial assessment and cross-checked the results. Discrepancies were discussed collaboratively, and if consensus could not be reached, a third reviewer with over 5 years of experience in evidence-based methodology and clinical practice was consulted to resolve disagreements. Inclusion was guided by the principles of evidence-based practice, prioritizing high-quality evidence and recently published authoritative literature.

#### 2.3.2 Quality assessment tools

Guidelines: clinical practice guidelines were evaluated using the Appraisal of Guidelines for Research and Evaluation II (AGREE II) instrument, which assesses multiple domains: scope and purpose, stakeholder involvement, rigor of development, clarity of presentation, applicability, and editorial independence. The tool comprises 23 individual items and two overall assessment items, each scored on a 7-point scale (1 = strongly disagree, 7 = strongly agree), with higher scores indicating better adherence. Standardized domain scores were calculated using the formula: standardized score (%) = (Obtained score – Minimum possible score) / (Maximum possible score – Minimum possible score) × 100%, Scores >60% were classified as Grade A, scores >30% and ≤ 60% as Grade B, and scores ≤ 30% as Grade C.

Systematic reviews, meta-analyses, and randomized controlled trials: these studies were evaluated using the Joanna Briggs Institute (JBI) Critical Appraisal Tools for Systematic Reviews and Research (2016 edition), which include 11 items covering the formulation of the evidence-based question, search strategy, quality appraisal of included studies, data extraction and synthesis, synthesis methods, and assessment of publication bias. Each item was assessed as “Yes,” “No,” “Unclear,” or “Not Applicable.” After group discussion, items marked as “No,” “Unclear,” or “Not Applicable” were further reviewed to determine inclusion, exclusion, or the need for additional information.

All quality assessments were independently conducted by two evidence-based practice–trained researchers. Any discrepancies were resolved through discussion or, if necessary, consultation with a third expert in evidence-based nursing to achieve consensus.

#### 2.3.3 Evidence extraction and synthesis

Given anticipated clinical and methodological heterogeneity across the included studies (e.g., differences in intervention modalities, follow-up durations, outcome measures, and patient populations), we did not perform a quantitative meta-analysis. Instead, we conducted a narrative evidence synthesis following JBI guidance. To manage heterogeneity, we grouped evidence by intervention type and outcome domain, and explicitly noted areas of inconsistency in the results.

Following the application of the inclusion and exclusion criteria, data were independently extracted by two nursing researchers trained in evidence-based practice and cross-checked to ensure accuracy and consistency. Extracted information included authors, publication year, source, study design, title, and level of evidence. The extracted data were subsequently reviewed and synthesized by the two researchers. When similar findings were identified across multiple sources, priority was given to evidence that was more directly relevant to patients with coronary artery disease and easier to apply in clinical practice. To minimize redundancy from overlapping evidence sources (e.g., when the same primary study was included in both a systematic review and an RCT pool), we applied a hierarchical priority rule: when a finding was supported by both a high-level source (guideline or systematic review) and primary studies, we preferentially extracted and cited the higher-level source. Primary studies were used only when higher-level evidence was unavailable or to illustrate specific intervention details not captured in existing syntheses. In cases of inconsistency, preference was given to evidence with higher methodological quality, more recent publication, and greater clinical authority. All included studies were graded according to the Joanna Briggs Institute (JBI) Levels of Evidence and Recommendation system. Evidence levels ranged from Level 1 to Level 5, with Level 1 representing the highest level of evidence. Specifically, in this summary, guidelines, systematic reviews, and meta-analyses were assigned Level 1; randomized controlled trials were assigned Level 2; and quasi-experimental studies were assigned Level 3. Recommendations were further classified according to effectiveness, feasibility, appropriateness, and clinical significance, and were categorized as Grade A (strong recommendation) or Grade B (weak recommendation).

## 3 Results

### 3.1 Literature search and characteristics of included studies

The initial search yielded 5,312 records. After removing duplicates using EndNote, 4,754 records remained. Screening of titles and abstracts resulted in 368 potentially relevant articles, which were subsequently assessed in full text. Finally, 38 studies met the inclusion criteria, including six guidelines, 13 systematic reviews, 15 randomized controlled trials, and four quasi-experimental studies. The characteristics of the included studies are summarized in Table 1.

**Table 1:** Characteristics of included studies.

No.	Included literature	Year	Literature sources	Type of evidence	Topic of the literature
1	Arnett et al. (22)	2019	Web of science	Guideline	2019 ACC/AHA guideline on the primary prevention of cardiovascular disease
2	Schwaab et al. (23)	2021	Embase	Guideline	Cardiac rehabilitation in German speaking countries of Europe—evidence-based guidelines from Germany, Austria and Switzerland LLKardReha-DACH—part 2
3	Chinese medical association (24)	2024	Yimai Tong	Guideline	Evidence-based practice guidelines for cardiac rehabilitation in coronary heart disease in China (2024 edition): part II
4	Chinese medical association et al. (25)	2020	Yimai Tong	Guideline	Primary care guidelines for cardiac rehabilitation in patients with coronary heart disease (2020)
5	Chinese society of gerontology and geriatrics (26)	2023	Yimai Tong	Guideline	Guidelines for chronic disease management in older adults with coronary heart disease
6	Zhu et al. (27)	2025	Yimai Tong	Guideline	Guidelines for self-health management in patients with chronic cardiovascular diseases
7	Douma et al. (28)	2024	PubMed	Systematic review	Effectiveness of behavior change techniques in eHealth-based cardiac rehabilitation in patients with coronary artery disease: a systematic review effective behavior change techniques in eHealth CR
8	Palacios et al. (29)	2017	Web of science	Systematic review	Internet-delivered self-management support for improving coronary heart disease and self-management-related outcomes a systematic review
9	Dale et al. (30)	2015	Web of science	Systematic review	The effectiveness of mobile-health behavior change interventions for cardiovascular disease self-management: a systematic review
10	He et al. (31)	2025	Web of science	Systematic review	The effectiveness of nurse-led tele-interventions on lipoprotein, blood pressure, self-efficacy, anxiety, and depression for patients with coronary heart disease: a short-term systematic review and meta-analysis
11	Su et al. (32)	2020	Web of Science	Systematic review	Effect of eHealth cardiac rehabilitation on health outcomes of coronary heart disease patients: a systematic review and meta-analysis
12	Kavradim et al. (33)	2020	Web of Science	Systematic review	Effectiveness of telehealth interventions as a part of secondary prevention in coronary artery disease: a systematic review and meta-analysis
13	Li et al. (34)	2025	Web of Science	Systematic review	Effectiveness of home-based cardiac rehabilitation interventions delivered via mHealth technologies: a systematic review and meta-analysis
14	Deng et al. (35)	2022	PubMed	Systematic review	The effect of telemedicine on secondary prevention of atherosclerotic cardiovascular disease: a systematic review and meta-analysis
15	Dwiputra et al. (36)	2023	Embase	Systematic review	Smartphone-based cardiac rehabilitation program improves functional capacity in coronary heart disease patients: a systematic review and meta-analysis
16	Zhu et al. (37)	2024	Embase	Systematic review	Effectiveness of mobile health applications on clinical outcomes and health behaviors in patients with coronary heart disease: a systematic review and meta-analysis
17	Sayli et al. (38)	2022	CINAHL	meta	Digital health interventions in patient management following acute coronary syndrome: a meta-analysis of the literature
18	Kamaruddin al (39)	2023	PubMed	meta	A meta-analysis of eHealth interventions on Ischaemic heart disease health outcomes
19	Wang et al. (40)	2025	Wanfang	meta	Effects of mobile app–based out-of-hospital health management interventions on physical and mental health and quality of life in patients with coronary heart disease: a meta-analysis
20	Keivanlou, (41)	2025	Web of Science	RCT	The effect of education using the interactive avatar application on self-care and the ability to identify and respond to the symptoms of heart attack in patients with acute coronary syndrome: a randomized clinical trial
21	Bae et al. (42)	2021	Web of Science	RCT	mHealth interventions for lifestyle and risk factor modification in coronary heart disease: randomized controlled trial
22	Hong et al. (43)	2021	Web of Science	RCT	Effectiveness of theory-based health information technology interventions on coronary artery disease self-management behavior: a clinical randomized waitlist-controlled trial
23	Pfaeffli Dale et al. (44)	2015	PubMed	RCT	Text message and internet support for coronary heart disease self-management: results from the Text4Heart randomized controlled trial
24	Li et al. (45)	2022	PubMed	RCT	Effects on adherence of a mobile application based self-management digital therapeutics among coronary heart disease patients: pilot randomized controlled trial
25	Keramat Kar et al. (46)	2023	PubMed	RCT	Effects of mobile health on self-care in senior patients with myocardial infarction
26	Kaihara et al. (47)	2023	Embase	RCT	The impact of dietary education and counseling with a smartphone application on secondary prevention of coronary artery disease: a randomized controlled study (the TeleDiet study)
27	Yu et al. (48)	2020	Cochrane Library	RCT	Smartphone-based application to improve medication adherence in patients after surgical coronary revascularization
28	Li et al. (49)	2025	Cochrane Library	RCT	i-CARE: a smartphone-based intervention to enhance cardiac rehabilitation in coronary artery disease—a randomized controlled trial
29	Su et al. (50)	2021	Cochrane Library	RCT	Effects of a nurse-led eHealth cardiac rehabilitation programme on health outcomes of patients with coronary heart disease: a randomized controlled trial
30	Yang (51)	2022	Wanfang	RCT	Effects of WeChat-based health education on self-management in patients with coronary heart disease after PCI
31	Lin et al. (52)	2024	Wanfang	RCT	Mobile health intervention based on the integrated theory of health behavior change in patients after PCI
32	Wang et al. (53)	2024	Wanfang	RCT	Mobile platform–based continuity of care in older patients with UnsTable Angina
33	Li et al. (54)	2022	VIP	RCT	Home-based tele rehabilitation for patients with coronary heart disease after PCI
34	Chen et al. (55)	2025	CNKI	RCT	Wearable device–based chronic disease management in patients after PCI
35	Yao et al. (56)	2021	CNKI	Quasi-experimental study	Effects of mobile app–based management on self-management and exercise capacity in patients with coronary heart disease after PCI
36	Pan et al. (57)	2018	Wanfang	Quasi-experimental study	Mobile health app–based self-management in patients undergoing percutaneous coronary intervention
37	Chen et al. (58)	2021	Wanfang	Quasi-experimental study	WeChat-based health education based on the health belief model in patients with coronary heart disease
38	Marvel et al. (59)	2021	Cochrane library	Quasi-experimental study	Digital health intervention in acute myocardial infarction

### 3.2 Quality assessment of included studies

#### 3.2.1 Guidelines

A total of six guidelines (22–27) were included in this study. All guidelines were rated as Grade B or above, with two receiving a Grade A rating and four receiving Grade B. The detailed quality assessment results are presented in Table 2.

**Table 2:** Quality assessment of guidelines.

Guideline	Percentage standardization by area (%)						≥60%	30%−60%	Recommended level
	①	②	③	④	⑤	⑥			
Donna et al. (22)	94.44	94.44	96.81	94.44	62.5	97.92	6	0	A
Schwaab et al. (23)	88.89	80.56	85.11	94.44	58.33	79.17	5	1	B
Chinese Medical Association. (24)	94.44	63.89	74.47	88.89	62.5	70.83	6	0	A
Chinese Medical Association et al. (25)	88.89	72.22	40.43	88.89	58.33	66.67	4	2	B
Chinese society of gerontology and geriatrics. (26)	94.44	63.89	39.36	80.56	39.58	33.33	3	3	B
Zhu et al. (27)	91.67	80.56	67.02	88.89	52.08	62.5	5	1	B

①Scope and purpose;

②Stakeholder involvement;

③Rigor of development;

④Clarity and presentation; and

⑤Applicability;

⑥Editorial independence.

#### 3.2.2 Systematic reviews

Ten systematic reviews (28–37) and three meta-analyses (38–40) were included. These studies primarily focused on the impact of eHealth, mobile health (mHealth), or telehealth interventions on self-management, functional capacity, and clinical outcomes in patients with coronary artery disease (28, 30, 36). Some studies specifically examined nurse-led teleinterventions, home-based cardiac rehabilitation, and telemedicine in secondary prevention (31, 34, 35), while others analyzed the effects of mobile health applications on patients' health behaviors and clinical indicators (37, 40). The results of the quality assessment are summarized in Table 3.

**Table 3:** Systematic evaluation or meta-analysis evaluation results.

Inclusion of literature	①	②	③	④	⑤	⑥	⑦	⑧	⑨	⑩	⑪
Douma et al. (28)	Yes	Yes	Yes	Yes	Yes	Yes	Yes	Yes	Unclear	Yes	Yes
Palacios et al. (29)	Yes	Yes	Yes	Unclear	Yes	Unclear	Yes	Yes	No	Yes	Yes
Dale et al. (30)	Yes	Yes	Yes	Unclear	Yes	Unclear	Yes	Yes	No	Yes	Yes
He et al. (31)	Yes	Yes	Yes	Yes	Yes	Yes	Yes	Yes	No	Yes	Yes
Su et al. (32)	Yes	Yes	Yes	Yes	Yes	Yes	Yes	Yes	No	Yes	Yes
Kavradim et al. (33)	Yes	Yes	Yes	Yes	Yes	Yes	Yes	Yes	Yes	Yes	Yes
Li et al. (34)	Yes	Yes	Yes	Yes	Yes	Yes	Yes	Yes	No	Yes	Yes
Deng et al. (35)	Yes	Yes	Yes	Yes	Yes	Yes	Yes	Yes	Yes	Yes	Yes
Dwiputra et al. (36)	Yes	Yes	Yes	Yes	Yes	Yes	Unclear	Yes	No	Yes	Yes
Zhu et al. (37)	Yes	Yes	Yes	Yes	Yes	Yes	Yes	Yes	Yes	Yes	Yes
Sayli et al. (38)	Yes	Yes	Yes	Yes	Yes	Yes	Unclear	Yes	No	Yes	Yes
Kamaruddin et al. (39)	Yes	Yes	Yes	Yes	Yes	Yes	Yes	Yes	Yes	Yes	Yes
Wang et al. (40)	Yes	Yes	Yes	Yes	Yes	Yes	Unclear	Yes	Yes	Yes	Yes

①Did the research questions and inclusion criteria for the review include the components of PICO?

②Whether the literature inclusion criteria were appropriate for that evidence-based question;

③Whether the search strategy used was appropriate;

④Whether the sources of the research papers were appropriate;

⑤Whether the criteria used to evaluate the quality of the literature were appropriate;

⑥Whether the evaluation of the quality of the literature was done independently by two or more evaluators;

⑦Whether the data were extracted with measures to reduce errors;

⑧Whether the methods used to synthesize/combine studies were appropriate;

⑨Whether possible publication bias was assessed;

⑩Whether recommendations for policy and/or practice were made with the support of the reported data; and

⑪Whether appropriate recommendations were made for future directions for further research.

#### 3.2.3 Randomized controlled trials

Fifteen randomized controlled trials (RCTs) (41–55) were included. Several studies investigated interactive virtual avatar education, mobile health, or smartphone applications targeting lifestyle modification, self-management, and medication adherence in patients with coronary artery disease (41, 42, 45). Other studies explored interventions delivered via WeChat platforms, cloud-based management systems, or SMS combined with internet support (44). Additional studies focused on nurse-led eHealth rehabilitation, theory-driven behavioral interventions, wearable devices, and home-based remote rehabilitation platforms for post-percutaneous coronary intervention (PCI) patients (50, 52, 54, 55). Quality assessment results are presented in Table 4.

**Table 4:** Results of quality evaluation of randomized controlled trials.

Inclusion of literature	①	②	③	④	⑤	⑥	⑦	⑧	⑨	⑩	⑪	⑫	⑬
Keivanlou (41)	Yes	No	Yes	No	No	Yes	No	Yes	Yes	Yes	Yes	Yes	Yes
Bae et al. (42)	Yes	Yes	Yes	No	N/A	Yes	Yes	Yes	Yes	Yes	Yes	Yes	Yes
Hong et al. (43)	Yes	Yes	Yes	No	No	No	No	Yes	Yes	Yes	Yes	Yes	Yes
Dale et al. (44)	Yes	Unclear	Yes	No	N/A	Yes	Unclear	Yes	Yes	Unclear	Yes	Yes	Yes
Li et al. (45)	Yes	Unclear	Yes	No	No	Yes	Unclear	Yes	Yes	Yes	Yes	Yes	Yes
Keramat Kar et al. (46)	Yes	Unclear	Yes	No	Unclear	Yes	Unclear	Yes	Yes	Unclear	Yes	Yes	Yes
Kaihara et al. (47)	Yes	Unclear	Yes	No	No	Yes	Unclear	Yes	Yes	Yes	Yes	Yes	Yes
Yu et al. (48)	Yes	Unclear	Yes	No	No	Yes	No	Yes	Yes	Yes	Yes	Yes	Yes
Li et al. (49)	Yes	Unclear	Yes	No	Unclear	Yes	Yes	Yes	Yes	Yes	Yes	Yes	Yes
Su et al. (50)	Yes	Yes	Yes	No	No	No	Yes	Yes	Yes	Yes	Yes	Yes	Yes
Yang (51)	Yes	Unclear	Yes	No	No	Yes	Unclear	Yes	Yes	Unclear	Yes	Yes	Yes
Lin et al. (52)	Yes	Unclear	Yes	No	No	Yes	Unclear	Yes	Yes	Unclear	Yes	Yes	Yes
Wang et al. (53)	Yes	Unclear	Yes	No	No	Yes	Unclear	Yes	Yes	Unclear	Yes	Yes	Yes
Li et al. (54)	Yes	Unclear	Yes	No	No	Unclear	Unclear	Yes	Yes	Yes	Yes	Yes	Yes
Chen et al. (55)	Yes	Unclear	Yes	No	No	Yes	Unclear	Yes	Yes	Unclear	Yes	Yes	Yes

①Whether to truly use random grouping;

②Whether to achieve hidden allocation;

③Is the baseline between groups comparable;

④Whether blinding was applied to the research subjects;

⑤Whether to implement blinding for the interveners;

⑥Whether blinding is applied to the evaluators of the results;

⑦Are the other measures received by each group the same, except for the intervention measures to be verified;

⑧Whether the follow-up is complete and whether measures have been taken to deal with lost follow-up;

⑨Will all randomly assigned research subjects be included in the analysis of the results;

⑩Whether to use the same method to evaluate the outcome indicators of each group of research subjects;

⑪Is the evaluation method for outcome indicators appropriate;

⑫Is the data analysis method appropriate; and

⑬Whether the research design is reasonable.

#### 3.2.4 Quasi-experimental studies

Four quasi-experimental studies (56–59) were included. Three of these investigated the effects of mobile applications on self-management in post-PCI coronary artery disease patients (56–58), while one study evaluated digital health interventions in patients with acute myocardial infarction (59). The quality assessment results are summarized in Table 5.

**Table 5:** Quality evaluation results of quasi-experiments.

Inclusion of literature	①	②	③	④	⑤	⑥	⑦	⑧	⑨
Yao et al. (56)	Yes	Yes	Yes	Yes	Unclear	Yes	Unclear	Unclear	Yes
Pan et al. (57)	Yes	Yes	Yes	Yes	Yes	Yes	Yes	Yes	Yes
Chen et al. (58)	Yes	Yes	Yes	Yes	Yes	Yes	Yes	Unclear	Yes
Marvel et al. (59)	Yes	Yes	Yes	Yes	Yes	Yes	Yes	Yes	Yes

①Has the causal relationship in the study been clearly explained;

②Is the baseline between groups comparable;

③Are the other measures received by each group the same, except for the intervention measures to be verified;

④Has a control group been established;

⑤Whether to implement diversified measurement of outcome indicators before and after the intervention;

⑥Is the follow-up complete? If incomplete, have you reported the loss of follow-up and taken measures to address the issue;

⑦Whether to use the same method to evaluate the outcome indicators of each group of research subjects;

⑧Is the evaluation method for outcome indicators reliable;

⑨And is the data analysis method appropriate.

### 3.3 Evidence summary

Where multiple evidence sources addressed the same recommendation, we prioritized the highest-level evidence and avoided redundant citation of all primary studies. Through systematic extraction and synthesis, the best available evidence on digital health interventions for self-management in patients with coronary artery disease was summarized into 36 evidence items across five domains: intervention modalities, self-management strategies, medication adherence, lifestyle management, and clinical outcomes. Detailed evidence items are presented in Table 6.

**Table 6:** Evidence summary of digital health interventions.

Category	Evidence statement	Evidence level	Grade of recommendation
Intervention forms	1. For older adults with coronary artery disease, patient registries should be established at the community level. Information technologies such as the internet, mobile applications, and WeChat platforms can be utilized to record self-management behaviors and facilitate the implementation of rehabilitation management and health interventions (26).	1	B
	2. Smartphone-based telehealth interventions have demonstrated significant effectiveness in improving key clinical outcomes in patients with coronary artery disease (35, 36).	1	B
	3. Short message service (SMS) is commonly used as a primary or supplementary intervention strategy, sometimes combined with web-based platforms, remote monitoring systems, or smartphone applications to support patient management (30, 31).	1	B
	4. Mobile health applications integrated with wearable devices, SMS services, real-time interaction, and social media platforms can provide multifaceted remote self-management support for patients with coronary artery disease, often grounded in behavior change theories (28, 36, 37).	1	A
	5. Digital wearable devices, such as smartwatches, can be used to collect health-related information during interventions. These devices may integrate monitoring tools including blood pressure monitors, heart rate trackers, blood glucose or lipid measurement devices, and mobile electrocardiogram recorders (28, 38).	1	A
	6. Interventions are typically delivered by multidisciplinary healthcare teams composed of healthcare professionals, including physicians or cardiologists, dietitians, psychological counselors, specialized nurses, and technical support staff (37).	1	A
Self-management intervention recommendations	7. Digital health interventions are recommended to incorporate a comprehensive empowerment strategy based on social cognitive theory, including individualized assessment, goal setting, action planning, offering choices, self-monitoring, and real-time feedback, to promote health behavior change in patients (29, 32).	1	B
	8. To improve medication adherence, behavior change techniques such as problem-solving, action planning, behavioral feedback, and self-monitoring of behavioral outcomes are suggested (28).	1	A
	9. Mobile application–based out-of-hospital health management interventions should encompass medication management, home-based self-management, and follow-up management to support secondary prevention in patients with coronary artery disease (40).	1	A
	10. Telehealth interventions are recommended to employ a combination of modalities, including phone calls, SMS, combined phone + SMS, and remote monitoring, to enhance the effectiveness of secondary prevention for coronary artery disease (33).	1	B
	11. Applications should include health education and structured courses, push notifications and reminders via SMS, and real-time interactive features to increase patient engagement (34, 37).	1	A
	12. For older adult populations, interventions should take into account the acceptability of novel eHealth technologies, as well as potential limitations in memory and hearing, and select more accessible modalities such as SMS, WeChat, or email (39).	1	A
	13. Applications should provide functionalities for medication management, measurement and exercise reminders, patient management, and guidance regarding medication adherence, medication-related issues, or adverse drug events (31, 40).	1	A
	14. Mobile applications are recommended to collect data on physical activity, blood pressure, heart rate, electrocardiogram readings, body weight, and self-reported symptoms for healthcare professional review, while simultaneously assisting patients in medication management and delivering information on ischemic heart disease risk factors via phone or smartwatch (38).	1	A
	15. Telephone or online access should be provided to enable patients to contact healthcare professionals post-discharge, allowing timely self-care actions during early disease deterioration and preventing avoidable readmissions (32).	1	B
	16. Mobile application–based out-of-hospital interventions are recommended to integrate techniques such as online gratitude activities, positive emotion journaling, social support, interactive communication, and leveraging individual strengths, along with timely psychological risk screening and monitoring, to prevent and improve anxiety and depression and enhance positive emotional states (40).	1	A
	17. Interventions should include additional components such as health education, nutritional advice, medication adherence support, psychosocial support, and smoking cessation guidance to comprehensively support patient self-management (31, 34).	1	B
	18. Applications are recommended to offer personalized settings, including goal setting, prescription planning, reminders, and feedback, to meet individualized patient needs (32, 37).	1	A
Medication adherence	19. Patients can utilize supportive tools to receive reminders for timely medication intake and to record information such as drug dosage, combination therapy, and follow-up monitoring schedules (27).	1	B
	20. Long-term use of aspirin, statins, and β-blockers is recommended for all patients with chronic coronary artery disease, with ongoing monitoring of bleeding events, heart rate, blood pressure, and related clinical parameters during therapy (27).	1	A
	21. Combining health education with mobile or tablet-based tools for SMS reminders and smart calls is recommended to enhance patient adherence to pharmacological treatment (24, 38).	1	A
	22. Providing feedback on patients' medication-taking behavior through digital health tools has been shown to effectively improve medication adherence (28, 38).	1	A
	23. Digital self-monitoring of behavioral outcomes, such as patient-recorded blood pressure, can be used to assess and reinforce medication adherence (28).	1	A
	24. SMS-based interventions can significantly improve medication adherence in patients with coronary artery disease. These interventions include daily medication reminders and personalized text support, with high patient satisfaction (30, 35).	1	B
Lifestyle management	25. Patients are encouraged to actively engage in eHealth interventions under the guidance of healthcare professionals, integrating psychological interventions and digital tools to enhance self-management skills and improve mental health outcomes (26, 27).	1	B
	26. Patients with coronary artery disease should participate in stress management training, emotion regulation strategies, and mindfulness exercises to promote psychological wellbeing and improve quality of life (27).	1	A
	27. Maintaining a regular daily schedule and good sleep hygiene is recommended. Patients should identify sleep disturbances, optimize sleep quality, clarify causes of insomnia, and implement targeted interventions. When necessary, seeking individualized, comprehensive medical treatment is advised (27).	1	A
	28. Engaging in appropriate physical activity is recommended, with careful selection of exercise type and intensity. Aerobic exercise should be prioritized, complemented by other forms of physical training as suitable (23, 25–27).	1	B
	29. All patients who smoke should be strongly encouraged to quit and avoid exposure to any form of tobacco, including electronic cigarettes, both actively and passively (24, 26, 27).	1	A
	30. Patients are advised to adopt a balanced dietary pattern, emphasizing plant-based foods while following low-salt and low-fat principles tailored to their condition. Attention should be paid to meal timing and frequency, and for patients with obesity, caloric intake should be restricted appropriately (22, 24, 27).	1	B
Clinical outcomes	31. Digital health interventions have been shown to improve functional capacity, blood pressure, resting heart rate, depressive symptoms, and health-related quality of life in patients with myocardial infarction (28, 34).	1	A
	32. Nurse-led remote interventions can enhance low-density lipoprotein levels, systolic and diastolic blood pressure, and self-efficacy in patients with coronary artery disease (31).	1	A
	33. eHealth-based cardiac rehabilitation has been demonstrated to effectively increase physical activity duration, daily step count, and quality of life, while reducing hospital readmission rates in patients with coronary artery disease (32).	1	B
	34. Internet-based interventions can lead to significant reductions in body weight and the frequency of angina episodes in patients with coronary artery disease (29).	1	B
	35. Mobile health applications can significantly decrease the incidence of major adverse cardiac events, hospital readmissions, total cholesterol, triglycerides, waist circumference, and anxiety and depression scores, while markedly improving left ventricular ejection fraction, maximal oxygen uptake, and six-minute walking distance (37).	1	A
	36. Psychological interventions play a positive role in enhancing quality of life among patients with cardiovascular disease and are effective in alleviating symptoms of depression and anxiety (23, 29, 37).	1	B

Evidence levels follow the JBI hierarchy.

Level 1 includes guidelines, systematic reviews, and meta-analyses; Level 2 includes randomized controlled trials; Level 3 includes quasi-experimental studies.

All evidence items in this table are derived from Level 1 sources.

## 4 Discussion

We acknowledge substantial heterogeneity across the included 38 studies, including variations in digital intervention platforms (e.g., SMS-based, smartphone applications, wearable devices), follow-up durations (ranging from 4 weeks to 12 months), and outcome measurements. This heterogeneity limits direct comparisons and precludes a pooled quantitative synthesis. To address this, we stratified evidence by five domains and prioritized findings supported by multiple high-level evidence sources.

Overall, the present evidence synthesis indicates that digital health interventions represent a promising and increasingly important strategy for supporting self-management among patients with coronary artery disease. The analysis suggests a comprehensive synthesis of the best available evidence on digital health interventions aimed at enhancing self-management among patients with coronary artery disease (CAD). Through a rigorous and structured integration of existing evidence, the findings suggest that digital health technologies are associated with positive changes in self-management behaviors and surrogate outcomes in improving patients' self-management capacity, medication adherence, health-related behaviors, and certain clinical outcomes. Notably, the conclusions of this evidence synthesis are highly consistent with the current body of international literature. Specifically, digital health tools—including mobile health (mHealth) applications, telemedicine platforms, and wearable devices—have been widely reported to support long-term chronic disease management. These technologies enable continuous health monitoring, real-time feedback, and personalized guidance, which collectively contribute to improved patient engagement and more effective disease management strategies (36–38). However, compared with traditional face-to-face health education, the effectiveness of digital health interventions may vary across different populations. Their impact is influenced by several factors, including patient age, digital health literacy, and accessibility to technological resources (39). Consequently, by systematically integrating multiple types of evidence, this study provides a balanced and evidence-based evaluation of digital health interventions in CAD self-management. Overall, the findings highlight the important role of digital health technologies in supporting self-management among patients with coronary artery disease and provide robust, hierarchical evidence to inform the implementation of digital health–supported strategies in clinical nursing practice.

### 4.1 Diversity of digital health interventions

This evidence summary synthesizes findings from multiple study designs, indicating that smartphone applications, SMS-based interventions, wearable devices, and remote monitoring platforms are the most widely applied digital health interventions for self-management in patients with coronary artery disease (CAD) (30, 31). Each modality has demonstrated efficacy in improving key self-management outcomes in CAD patients (35, 36, 43), confirming the clinical utility of digital health interventions in this population. Compared with traditional face-to-face health education, digital health platforms provide patients with continuous, convenient, and individualized support, effectively extending healthcare services from hospitals into community and home settings. Recent studies increasingly emphasize the importance of integrating multiple digital technologies, as single-modality interventions are gradually being replaced by multi-component strategies. For example, a smartphone application may be supplemented with SMS reminders, combined with web-based information and interactive features, while remote monitoring devices collect physiological data to inform personalized interventions that are subsequently communicated to patients via SMS feedback (30). A typical integrated model centers on a smartphone application, combined with SMS services for real-time reminders on medication adherence and rehabilitation exercises. Web platforms are used to deliver health education, facilitate patient-provider communication, and provide peer support. Simultaneously, wearable devices and portable monitoring tools collect real-time physiological data, including heart rate, blood pressure, physical activity duration, and sleep quality (47, 55). Healthcare professionals then synthesize multidimensional data to assess patient status and self-management behaviors, formulate individualized intervention plans, and deliver targeted guidance through application-based messaging or SMS, establishing a closed-loop management system. Moreover, structured digital interventions require implementation by a multidisciplinary team, including nurses, physicians, dietitians, and psychological counselors (37). Such collaboration ensures the comprehensiveness and personalization of interventions and is critical for improving long-term outcomes in CAD patients and achieving standardized management of cardiovascular chronic diseases.

### 4.2 Promotion of self-management capacity by digital health interventions

Self-management is a critical determinant of long-term outcomes in patients with coronary artery disease (CAD). Effective self-management encompasses symptom monitoring, adherence to prescribed medications, maintenance of a healthy lifestyle, and timely recognition of early signs of disease exacerbation (31, 34, 51). However, during post-discharge long-term management, many patients struggle to sustain consistent self-management behaviors due to insufficient disease knowledge, lack of continuous support, and low self-efficacy. The evidence synthesized in this review indicates that digital health interventions can significantly enhance self-management capacity in CAD patients (28, 29, 57). Digital platforms typically employ structured health education content, self-monitoring tools, and real-time feedback mechanisms to actively engage patients in disease management (29, 32, 37). The choice of intervention modality should consider the characteristics of the target patient population. For older adults, sensory or cognitive limitations may necessitate the use of more suitable interventions, such as SMS or WeChat-based communication (39). For post-percutaneous coronary intervention (PCI) patients, early rehabilitation behaviors and medication adherence should be prioritized (52). This “multi-component integration combined with individualized adaptation” approach provides a practical framework for the clinical implementation and promotion of digital health interventions.

Mechanistically, positive effects likely operate through three pathways: behavior change techniques (goal setting, self-monitoring, feedback) that enhance self-efficacy; patient empowerment through accessible health information; and continuous support via closed-loop monitoring. However, formal mediation analyses are lacking in most primary studies.

### 4.3 Impact on medication adherence

Medication adherence is a critical component of secondary prevention in patients with coronary artery disease (CAD). Long-term, guideline-directed use of cardiovascular protective medications, including antiplatelet agents, statins, and β-blockers, plays a pivotal role in reducing the risk of recurrent cardiovascular events (27). However, post-discharge, many patients demonstrate suboptimal adherence, which can compromise therapeutic outcomes. The evidence synthesized in this review indicates that digital health interventions can effectively improve medication adherence in CAD patients (30, 35, 38). Mobile applications and remote health management systems frequently incorporate medication reminder features, using automated prompts and electronic medication logs to support the development of consistent adherence behaviors (24, 38, 45, 59). In addition, interventions delivered via WeChat platforms, such as daily check-ins and blood pressure monitoring within patient groups, have been shown to enhance post-percutaneous coronary intervention (PCI) patients' adherence (48). Similarly, cloud-based health management systems achieve comparable outcomes, offering added clinical value in monitoring disease progression, preventing recurrence, and reducing mortality (22, 27). Collectively, these findings underscore the significant potential of digital health interventions in optimizing medication management among patients with CAD.

### 4.4 Support for lifestyle management through digital health interventions

Healthy lifestyle behaviors are a fundamental component of secondary prevention in patients with coronary artery disease (CAD). Clinical guidelines universally recommend adherence to balanced diets, regular physical activity, smoking cessation, alcohol moderation, and weight management. However, in real-world settings, many patients struggle to sustain these behaviors over time, which represents a key barrier to effective long-term disease management. A meta-analysis of 15 studies demonstrated that digital interventions significantly improve physical activity and exercise capacity: participants in the intervention groups exhibited higher levels of physical activity and exercise performance compared with controls. Regarding diet and lipid-related lifestyle behaviors, intervention participants showed significantly lower levels of low-density lipoprotein cholesterol, triglycerides, and total cholesterol, indicating that mobile application-based interventions can positively influence both exercise habits and diet-related behaviors (40). The 2020 Primary Care Guidelines for Coronary Heart Disease Cardiac Rehabilitation explicitly recommend the use of integrated digital models to support lifestyle and psychological health management, particularly in the post-discharge rehabilitation phase (25). Digital lifestyle management primarily relies on wearable devices, mobile applications, and cloud-based platforms to enable real-time monitoring and personalized guidance on patients' physical activity, diet, and sleep behaviors. A randomized controlled trial (RCT) targeting post-percutaneous coronary intervention (PCI) patients reported that wearable device-based chronic disease management led to a body mass index (BMI) compliance rate of 85% in the intervention group, significantly higher than 45% in the control group (55). Another RCT focusing on dietary education confirmed that digital tools substantially improved CAD patients' dietary habits and overall lifestyle behaviors (47). Additionally, social platforms, such as WeChat, can establish peer-support communities, facilitating mutual learning, reinforcing healthy behaviors, and improving self-management capacity and health-related quality of life among patients with stable CAD (51).

Digital psychological health interventions, delivered via mobile applications or remote counseling platforms, provide structured services including mental health risk screening, emotion regulation guidance, and mindfulness exercises. However, current evidence supporting internet-based interventions for emotional outcomes in CAD patients remains limited. Only targeted cognitive behavioral therapy (CBT) interventions for depression have demonstrated clear positive effects, whereas psychological support provided as an adjunct to lifestyle management shows minimal benefit (29). Given that depression and anxiety are independent predictors of adverse cardiovascular outcomes, the development of internet-based interventions targeting both psychological wellbeing and cardiac self-management is a critical priority. Future interventions should integrate evidence-based psychological therapies, such as CBT, and clearly articulate their theoretical underpinnings and mechanisms of action.

The integrated digital model enables synergistic, multidimensional interventions. Its major advantage lies in allowing nurses, as primary coordinators, to consolidate patients' medication, lifestyle, and psychological data via digital platforms, thereby naturally and systematically formulating individualized intervention plans (31).

### 4.5 Impact on clinical outcomes

Beyond improvements in behavioral and cognitive outcomes, a central question is whether digital health interventions can translate into measurable improvements in clinical outcomes. The evidence included in this review suggests that digital health interventions can, to some extent, reduce rehospitalization rates, enhance quality of life, and facilitate the management of cardiovascular risk factors (31). Multiple systematic reviews and meta-analyses indicate that nurse-led digital health interventions can improve low-density lipoprotein cholesterol (LDL-C), blood pressure, and self-efficacy in patients with coronary artery disease. However, statistically significant effects on high-density lipoprotein cholesterol (HDL-C), anxiety, and depression have not yet been consistently demonstrated (31). Notably, studies have shown that participants receiving digital interventions exhibit significantly higher left ventricular ejection fraction and lower left ventricular end-diastolic and end-systolic diameters compared with controls, suggesting that home-based remote cardiac rehabilitation platforms can support post-percutaneous coronary intervention (PCI) cardiac function recovery (54). Specific interventions have demonstrated clinically meaningful benefits: WeChat-based continuity-of-care programs reduced the incidence of adverse cardiac events in older patients from 25.49 to 7.84% within 6 months (53), while mobile application interventions lowered total cholesterol by 0.19 mmol/L and triglycerides by 0.24 mmol/L (37). The potential mechanisms through which digital health interventions improve clinical outcomes include three main pathways: (1) Behavioral theory-based self-efficacy enhancement: interventions grounded in health behavior change theories increase patient self-efficacy, promoting the adoption and long-term maintenance of healthy behaviors, which in turn strengthens self-management capacity (28). However, no included study formally tested mediation through self-efficacy. (2) Continuity of care from hospital to home: digital platforms facilitate ongoing monitoring and professional guidance post-discharge, addressing the limitations of conventional face-to-face care. Nurse-led remote interventions significantly improve continuity-of-care scores (50). Yet, the added benefit beyond standard telephone follow-up remains unclear. (3) Integrated, real-time data monitoring for individualized intervention: by combining medication adherence, lifestyle, psychological, and physiological data, digital systems can enable precise evaluation and tailored interventions. But causal attribution is limited because most studies combined multiple components. When adherence declines or abnormal physiological indicators emerge, the system can automatically alert healthcare providers to intervene proactively, thereby preventing adverse event (30).

Nevertheless, current evidence regarding the long-term effects of digital health interventions on clinical outcomes is heterogeneous, with variations in intervention modalities, duration, and patient populations, limiting direct comparisons. Therefore, future research should focus on high-quality, long-term randomized controlled trials to systematically evaluate the sustained clinical impact of digital health interventions in the management of coronary artery disease.

A critical gap in the current evidence base deserves explicit acknowledgment. Among the 38 included studies, the vast majority reported behavioral or surrogate outcomes—such as medication adherence scores, physical activity duration, step counts, self-reported dietary habits, blood pressure, and lipid levels—rather than hard cardiovascular endpoints. Only three RCTs reported major adverse cardiovascular events (MACE) as a secondary outcome, and none were adequately powered to detect differences in all-cause mortality or cardiovascular death (48–50). Improvements in surrogate outcomes, while encouraging, do not automatically translate into reduced hard clinical endpoints. This gap between behavioral improvements and long-term prognosis is not unique to digital health research but is particularly relevant here because most intervention studies had short follow-up periods (median ≤ 6 months). Therefore, we caution readers against assuming that the observed improvements in self-management behaviors and surrogate markers necessarily predict reductions in MACE or mortality. Future trials should include long-term follow-up (≥12 months) and pre-specified hard clinical endpoints to establish the clinical value of digital health interventions.

### 4.6 Implications for nursing practice

The evidence synthesized in this review has significant implications for nursing practice. Nurses play an indispensable role in patient education, chronic disease management, and post-discharge follow-up for individuals with coronary artery disease. With the advancement of digital health technologies, nurses can remotely monitor and manage patients via online platforms. Digital health tools enable nurses to provide individualized guidance and behavioral interventions based on real-time patient data, thereby enhancing patients' self-management capabilities. Moreover, digital interventions maintain communication between patients and healthcare providers, reducing the need for in-person visits. Consequently, integrating digital health technologies with traditional nursing services is poised to become a key strategy for the future management of chronic cardiovascular conditions.

Factors influencing intervention effectiveness include patient age (older adults may prefer SMS over apps), digital health literacy, intervention adherence (rarely reported), and multidisciplinary team involvement. These variables were inconsistently collected across studies, limiting tailored recommendations.

## 5 Limitations

This study has several limitations. First, the lack of standardized definitions for “self-management” and “digital health interventions” may have resulted in incomplete retrieval of relevant literature; more refined search strategies are needed to expand coverage. Second, only English and Chinese language publications were included, which may have introduced selection bias and affected the comprehensiveness of results. Third, given the rapid evolution of digital technologies, the included studies may not fully reflect the most current interventions. Fourth, most randomized controlled trials were conducted in specific geographic regions, which may limit the external validity of the findings. Fifth, as an evidence summary following JBI methodology, this study does not generate new primary data or provide pooled quantitative effect estimates. Therefore, we cannot formally test for publication bias or heterogeneity, nor can we quantify the magnitude of benefit. Readers seeking effect sizes should refer to the individual meta-analyses included in this synthesis. Our summary provides a structured overview of the current evidence base, not a definitive causal assessment. Finally, the majority of included studies relied on surrogate or behavioral outcomes rather than hard clinical endpoints, and follow-up durations were generally short ( ≤ 6 months). Consequently, this evidence summary cannot draw conclusions about the impact of digital health interventions on mortality or major adverse cardiovascular events.

## 6 Conclusion

The findings of this evidence summary suggest that digital health interventions are associated with improvements in self-management behaviors, medication adherence, and certain surrogate clinical outcomes (e.g., blood pressure, LDL-cholesterol) in patients with coronary artery disease. However, evidence linking these improvements to hard cardiovascular endpoints—such as mortality, major adverse cardiovascular events, or unplanned rehospitalizations—remains limited due to short follow-up durations and the pre-dominance of surrogate outcomes in the included studies. Therefore, these findings should be interpreted as hypothesis-generating rather than practice-changing at this stage. Integrating digital technologies—such as mobile applications, digital devices, text messaging services, remote monitoring platforms, and wearable devices—into self-management programs may improve patient outcomes, enhance medication adherence, and promote healthy lifestyle behaviors. Future clinical practice should prioritize the implementation of digital interventions, with individualized designs tailored to patients' age, technological literacy, and preferences. High-quality, long-term randomized controlled trials are warranted to further validate the effectiveness of digital interventions and provide robust evidence for the digital management of coronary artery disease.

## Data Availability

The original contributions presented in the study are included in the article/supplementary material, further inquiries can be directed to the corresponding authors.
